# Magnetron-Sputtered, Biodegradable FeMn Foils: The Influence of Manganese Content on Microstructure, Mechanical, Corrosion, and Magnetic Properties

**DOI:** 10.3390/ma11040482

**Published:** 2018-03-23

**Authors:** Till Jurgeleit, Lea Katharina Jessen, Eckhard Quandt, Christiane Zamponi

**Affiliations:** Institute for Materials Science, Faculty of Engineering, University of Kiel, Kaiserstrasse 2, 24143 Kiel, Germany; leje@tf.uni-kiel.de (L.K.J.); eq@tf.uni-kiel.de (E.Q.)

**Keywords:** magnetron sputtering, biodegradable metals, FeMn alloys, material characterization, mechanical properties, corrosion properties, magnetic properties

## Abstract

FeMn alloys show a great potential for the use as a biodegradable material for medical vascular implants. To optimize the material properties, with respect to the intended application, new fabrication methods also have to be investigated. In this work different Fe–FeMn32 multilayer films were deposited by magnetron sputtering. The deposition was done on a substrate structured by UV lithography. This technique allows the fabrication of in-situ structured foils. In order to investigate the influence of the Mn content on the material properties foils with an overall Mn content of 5, 10, 15, and 17 wt % were fabricated. The freestanding foils were annealed post-deposition, in order to homogenize them and adjust the material properties. The material was characterized in terms of microstructure, corrosion, mechanical, and magnetic properties using X-ray diffraction, electron microscopy, electrochemical polarization, immersion tests, uniaxial tensile tests, and vibrating sample magnetometry. Due to the unique microstructure that can be achieved by the fabrication via magnetron sputtering, the annealed foils showed a high mechanical yield strength (686–926 MPa) and tensile strength (712–1147 MPa). Owing the stabilization of the non-ferromagnetic ε- and γ-phase, it was shown that even Mn concentrations of 15–17 wt % are sufficient to distinctly enhance the magnetic resonance imaging (MRI) compatibility of FeMn alloys.

## 1. Introduction

Biodegradable metallic materials for the intended use as temporary implants (e.g., stents) are a topic of intense research. One of the most prominent material classes are iron-based materials. In early in vivo studies by Peuster et al. [1,2], it was shown that the general feasibility of Fe as a biodegradable material, with regards to its biocompatibility, is given. However, with respect to the desired degradation time of 6–12 months, the degradation rate of pure Fe was found to be too slow. Therefore, from the material science point of view, the goal is to increase the corrosion rate of the material. Another possible strategy is to enhance its strength, to the extent that thinner structures bear the load acting on an implant and relativize the low corrosion rate. A further drawback are the ferromagnetic (FM) properties of Fe, which can cause complications during magnetic resonance imaging (MRI), such as implant heating, torque, or image distortions due to susceptibility effects [3]. One strategy to obtain the desired material properties is alloying. One of the most promising alloying elements is Mn. Alike Fe, Mn is an essential trace element in the human metabolism [4]. Furthermore, FeMn alloys are known for their good mechanical properties. Since Mn exhibits a lower standard potential than Fe [5], the overall corrosion potential of the FeMn alloy is in turn shifted to lower values, and the alloy should be more prone to corrosion. Additionally, FeMn alloys with high Mn concentrations of 15–35 wt % are known for their high strength and even ductility. This is attributed to the two effects called transformation induced plasticity (TRIP) and twinning induced plasticity (TWIP) [6]. An additional advantage of those highly-alloyed FeMn alloys are the magnetic properties. FeMn alloys exist in three crystal modifications: the α′-, ε-, and γ-phases. While the FM α′-phase occurs at rather low Mn concentrations, the ε- and γ-phase are stable at higher Mn concentrations [7]. It was found that the ε-phase exhibits paramagnetic (PM) behavior, whereas the γ-phase shows antiferromagnetic (AFM) behavior [8,9], and thus is beneficial for biodegradable implant applications. Hermawan et al. first investigated FeMn alloys with 20, 25, 30, and 35 wt % Mn content. The alloys showed significantly higher strength and corrosion rates compared to pure iron. All the investigated alloys showed AFM behavior with an saturation magnetization below those of the stainless steel 316 L (SS 316 L), which acts as a gold standard for medical implants [10,11].

In previous works of the authors, it was shown that magnetron sputtering is a feasible method for fabricating freestanding micro-patterned devices for degradable implants. A high strength was observed, due to the unique microstructure achieved by this technique [12,13,14,15]. The usage of magnetron sputtering in combination of UV lithography for the fabrication of NiTi devices for medical applications was first shown by Zamponi, Siekmeyer, and de Miranda et al. [16,17,18,19]. Furthermore, the method was adapted by Schlüter and Haffner et al. for the fabrication of biodegradable Mg-based devices with feature sizes down to 5 μm [20,21,22,23]. In other work, the technique was used for the fabrication of biodegradable FeMn foils. It was found that due to the microstructure, the foils show a higher strength compared to the values of FeMn alloys with comparable compositions presented in literature. Furthermore, the saturation magnetization of the annealed foils was found to be lower compared to a SS 316 L reference [15].

Because there are worries about Mn toxicity, the daily dose should not exceed 500 μg/day [4,24,25]. Even though it is almost impossible to reach this limit with a vascular stent that degrades over 6–12 months, the Mn content should be as small as possible, but as high as necessary. Considering the non-equilibrium phase diagram for FeMn [26,27], the desired phase composition that only contains the ε- and γ-phase, and thus non-ferromagnetic behavior, can be expected at significantly lower Mn concentrations than the 20 wt % Mn reported by Hermawan [10]. Hence, in this study, the influence of different Mn concentrations (5, 10, 15, and 17 wt %) on the microstructure—and in turn the magnetic properties, mechanical behavior, and corrosion—was investigated. In order to achieve the desired composition, the foils were fabricated by sputtering Fe-FeMn32 multilayers (ML), followed by a heat treatment for homogenization. The magnetic characterization was done via vibrating sample magnetometry (VSM). Electrochemical polarization measurements and immersion tests were performed in order to evaluate the corrosion rates under in vitro conditions. The mechanical properties were determined by uniaxial tensile tests. The microstructural characterizations were done via X-ray diffraction (XRD), scanning electron microscopy (SEM), energy-dispersive X-ray spectroscopy (EDX), and focused ion beam (FIB) analysis.

## 2. Materials and Methods

### 2.1. Sample Preparation

**Figure 1:**
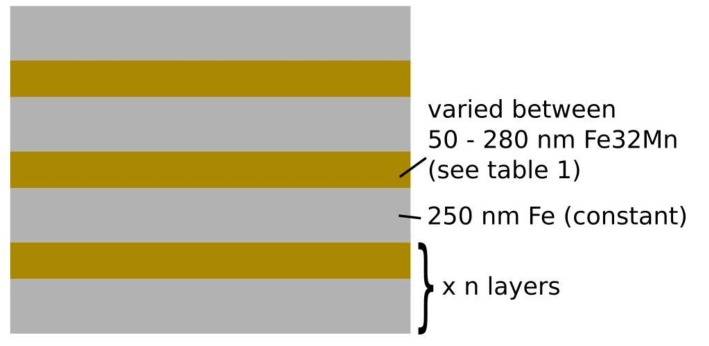
Schematic drawing of Fe–FeMn32 multilayer stack.

All sputter depositions in this work were done in a Von Ardenne CS730S (VON ARDENNE, Dresden, Germany) cluster magnetron sputtering machine. Crystalline, Z-cut, quartz wafers with 10 cm diameter were used as substrate material. Three different sample designs were used. Square shaped foils (15 mm × 15 mm) with a thickness of 10 μm were used for the corrosion tests and XRD measurements. For the tensile tests, “dog-bone” shaped samples with a strut width of 0.5 mm, 7 mm strut length, and a homogeneous thickness of 20 μm were used. The VSM measurements were performed on circular-shaped foils with a radius of 1 mm and 20 μm thickness. For more details about the structuring process, refer to previous work [12,14,15]. As sputter targets, a pure Fe plate with (20 cm diameter) (FHR, Ottendorf-Okrilla, Germany) and a Fe35Mn plate (10 cm diameter) (Ingpuls, Bochum, Germany) were used, both with a purity of 99.9%. All foils were deposited as multilayers, where the first and last layer was a Fe layer. A schematic drawing of a multilayer stack is shown in Figure 1. The Fe layer thickness was kept constant at 250 nm, whereas the Fe35Mn layer thickness varied between 50–280 nm. In this way, batches of different overall Mn concentrations (5, 10, 15, and 17 wt %) were fabricated. In the following, the different samples are named FeMnX. Here, X stands for the mean, overall Mn content ± 0.5 wt % in the foils determined by EDX, after the homogenization step explained below. Table 1 displays an overview of the sample names, corresponding desired Mn layer thickness, and the measured nominal Mn content. The multilayer stacks were released from the substrate by selective chemical wet etching of the sacrificial layer. In order to homogenize the foils and reduce the defect density, the samples were annealed. Based on the experience of previous work, samples were annealed at 800 °C and 950 °C for two hours. In order to prevent oxidation, the heat treatments were performed under a reducing atmosphere (Varigon H10^®^, Linde, Pullach, Germany), as described previously [15].

### 2.2. Microstructure

#### 2.2.1. X-ray Diffraction

The identification of the crystallographic phases were done using an X-ray diffractometer XRD-3000 PTS (Seifert, Ahrensburg, Germany), employing monochromatic Cu-Kα radiation. The θ–2θ absolute scans were performed in the range of 35° to 90°, with 0.05° step width and 3 s dwell time per step.

#### 2.2.2. Scanning Electron Microscopy/Energy-Dispersive X-ray Spectroscopy

Further investigations regarding the composition and microstructure were done by EDX and SEM. For the investigations of the composition, an SEM (Zeiss Ultra Plus, Oberkochen, Germany) associated with an EDX detector (Oxford instruments, Abingdon, UK) was used. Cross-sectional SEM images were prepared by FIB NanoLab 600 (FEI, Frankfurt, Germany) to obtain the grain structure. In order to reveal the grains, ion beam excitation was used for the images, to give an additional orientation contrast due to channeling effects. The grain size was optically measured.

### 2.3. Corrosion

#### 2.3.1. Electrochemical Polarization Measurements

Electrochemical, linear polarization corrosion measurements were performed as previously reported [12], according to the ASTM G59-97 [28]. As electrolyte, Hanks’ buffered salt solution (HBSS) (H1387 Sigma Aldrich, Taufkirchen, Germany) was modified with sodium bicarbonate at 0.35 g/L. The temperature of the solution was held constant at 37 ± 1 °C, and the pH was adjusted by CO_2_ inlet and regulated to 7.4 ± 0.05. The polarization curves were measured using a three-electrode cell and a VersaStat 3 (Princton Applied Research, Oak Ridge, TN, USA). A Pt mesh as counter electrode, an Ag/AgCl reference electrode, and the corrosion samples as working electrodes were used. The corrosion current density rates were determined by Tafel extrapolation [29,30], in order to calculate the corrosion rates (CR) using (Equation (1)):

(1) $$CR=\frac{j_{c}M}{n\rho F}$$

where *j*_c_ is the corrosion current density (A/m^2^), *ρ* is the density (7690 kg/m³), *M* is the molar mass (55 g/mol), *n* is the number of elementary charges per reaction step (2), and *F* is the Faraday constant. Based on investigations regarding the degradation mechanisms of Fe [31] and FeMn-based alloys [32], the anodic dissolution reaction follows (Equations (2) and (3)).

In order to determine the mean value and deviation, four samples of each type were measured.

(2) $$Fe\to Fe^{2+}+2e^{-}$$

(3) $$Mn\to Mn^{2+}+2e^{-}$$

#### 2.3.2. Immersion Tests

Additionally, immersion tests were performed to determine the weight loss. The test conditions were the same as described above. Before the tests, the weight of the samples was determined using a high accuracy balance, and samples were cleaned in isopropanol. All samples were immersed in 600 mL HBSS for 12 days. After the immersion period, the samples were washed in de-ionized (DI) water, and residual corrosion products were carefully removed by dipping them into diluted H_3_PO_4_ for 20 s. Next, the samples were carefully rinsed in DI water and isopropanol, and fast-dried with nitrogen. Finally, the weight loss (WL) was determined. Four samples of each type were measured.

### 2.4. Mechanical Properties

Uniaxial tensile tests were performed using the above described “dog-bone” shaped samples and a testing machine of the type BETA 5-5/6 × 10 (Messphysik, Fürstenfeld, Austria), with a special sample holder for thin samples. A straining rate of 0.4%/min was applied. For the fracture criterion, a force reduction of 60% relative to the maximum applied force was used. For each type, four samples were measured to assess the statistic reliability. Since for the desired application exclusively the annealed samples are of interest, no as-deposited samples were tested.

### 2.5. Magnetic Properties

A vibrating sample magnetometer (VSM) of the type Lake Shore 7400 series (Lake Shore, Darmstadt, Germany) was used to record magnetic polarization curves, in order to determine saturation polarization *J*_S_, remanence *J*_R_, and coercive field *H*_C_. The measurements were performed in-plane, with a range of ±0.5 T and with a ramping rate of 3 mT/s.

## 3. Results

### 3.1. Microstructure

#### 3.1.1. X-ray Diffraction

**Figure 2:**
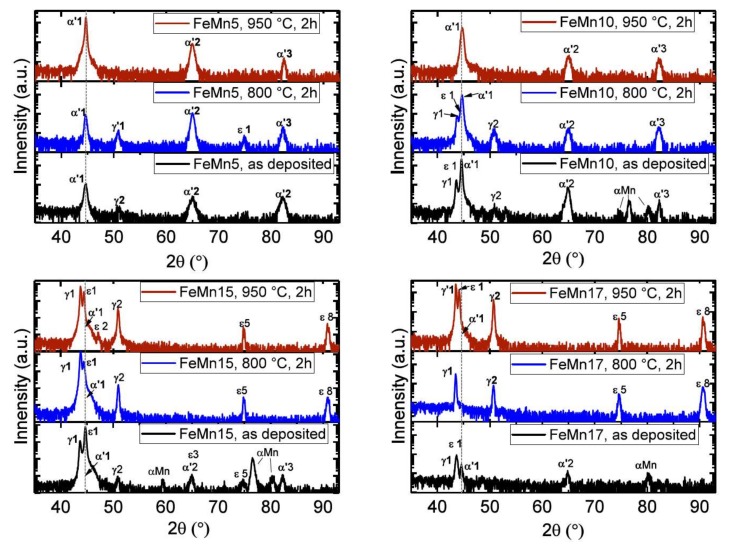
XRD measurements of different FeMnX samples in the as deposited and annealed state. The 2θ angles and lattice planes corresponding to the labeled reflexes are presented in Table 2.

In Figure 2, the XRD measurements of different FeMnX samples in the as-deposited and annealed state are shown. The measured reflexes are indexed by a Greek letter and a number, respectively. The letter refers to the phase type, and the number to the corresponding lattice plane. The indexes, and their corresponding 2θ values and lattice planes, are shown in Table 2. All as-deposited samples showed reflexes of the α′- and γ-phase. Furthermore, additional reflexes of the ε-phase and the α-Mn phase were observed in the samples with higher Mn concentrations (5–17 wt %). The annealed FeMn5 samples showed an increase of the intensity of the α′-phase reflexes. After annealing at 800 °C, reflexes of the γ- and ε-phase were also present. Only reflexes of the α′-phase were observed for the samples annealed at 950 °C. The measurement of the FeMn10 sample looks similar; however, the detected intensity of the γ reflexes was higher. The measurement of the annealed FeMn15/17 samples showed a different behavior. Here, annealing led to the reduction of the α′-phase reflexes, while reflexes of the γ- and ε-phase became more pronounced. The FeM15/17 samples did not even show any definite α′-phase reflexes in the annealed state. However, in general the reflexes were quite broad; therefore, the main α′1 reflex cannot be definitively excluded.

#### 3.1.2. Scanning Electron Microscopy

**Figure 3:**
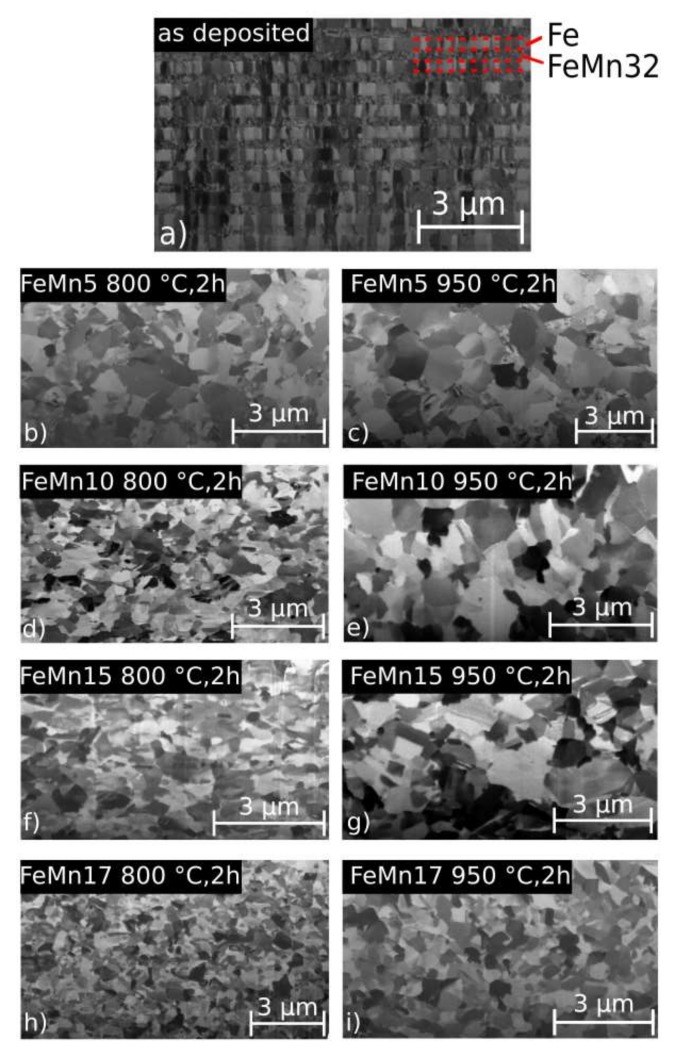
Cross-sectional SEM images of the microstructure: (**a**) shows the example as-deposited micro structure (FeMn15 sample). (**b**) shows FeMn5 at 800 °C, for 2 h; (**c**) FeMn5 at 950 °C, for 2 h; (**d**) FeMn10 at 800 °C, for 2 h; (**e**) FeMn10 at 950 °C, for 2 h; (**f**) FeMn15 at 800 °C, for 2 h; (**g**) FeMn15 at 950 °C, for 2 h; (**h**) FeMn17 at 800 °C, for 2 h; and (**i**) FeMn17 at 950 °C, for 2 h.

Figure 3 shows the cross-sectional SEM images used to determine the grain sizes of the samples. The grain size values are presented in Table 3. After annealing, there was no evidence for a layered structure like in the as deposited samples, exemplary shown for the FeMn15 sample in Figure 3a. Overall, the annealed samples showed a homogeneous fine-grained structure. The mean grain size was in the sub-μm range for the 800 °C as well as for the 950 °C annealed samples. In general, with higher annealing temperatures, larger grain size values were observed. Furthermore, there is a trend of decreasing grain size with increasing Mn content, independently of the annealing temperature.

### 3.2. Corrosion

**Figure 4:**
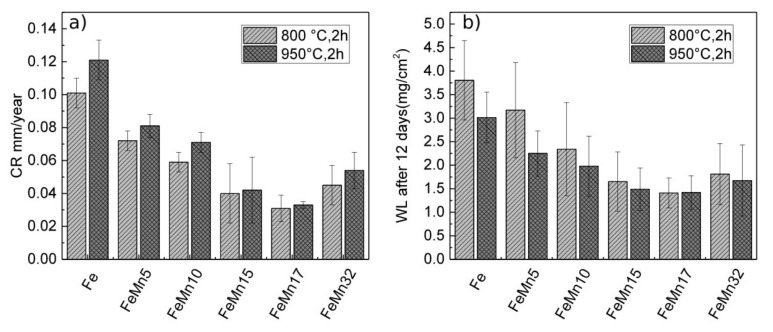
Graphically representation of (**a**) the mean corrosion rates and standard deviations determined from the electrochemical polarization measurements, and (**b**) the weight loss determined by immersion tests.

In Table 4 and Figure 4, the results of the corrosion measurements are presented. In addition, previously-presented values of pure Fe and FeMn32 [12,14,15] are given as a reference. The CR determined from the electrochemical measurements were decreasing with increasing Mn content (0–10 wt %) at first; this decrease is saturated with further increase of the Mn content (15–32 wt %) and remains constant within the error range. With lower Mn concentrations, a higher CR is found for the samples annealed at 950 °C. At higher Mn concentrations, there is no evidence for the influence of the annealing temperature on the CR.

### 3.3. Mechanical Properties

**Figure 5:**
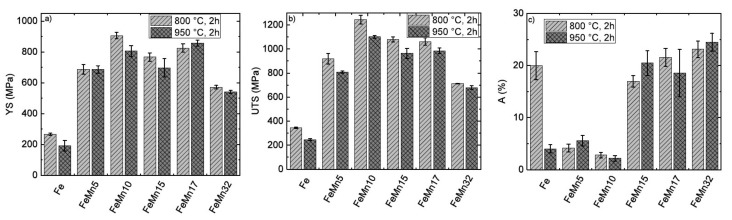
Graphically depiction of the (**a**) yield strength (YS), (**b**) ultimate tensile strength (UTS) and (**c**) fracture strain (A) for the different FeMnX samples, after annealing at 800 °C and 950 °C for 2 hours. Additionally, the values of pure Fe [12] and FeMn32 are shown [15].

Figure 5 shows the graphical depiction of fractures strain (A), yield strength (YS), and ultimate tensile strength (UTS) of all samples. The values are presented in Table 5. Additionally, the values for pure Fe [12] and FeMn32 are given as a reference. At low Mn concentrations (5 wt %, 10 wt %), the fracture strain was significantly smaller compared to that of pure Fe. The fracture strain of the FeMn10 samples was slightly lower compared to that of the FeMn5 samples. The YS and UTS of the FeMnX samples was strongly increased compared to those of pure Fe. The strength increased with the Mn content up to 10 wt %, and slightly decreased at higher Mn concentrations (>10 wt %). The YS of the FeMn17 sample was higher compared to the YS of FeMn15 for both annealing temperatures, whereas the UTS was almost the same for both sample types.

### 3.4. Magnetic Properties

**Figure 6:**
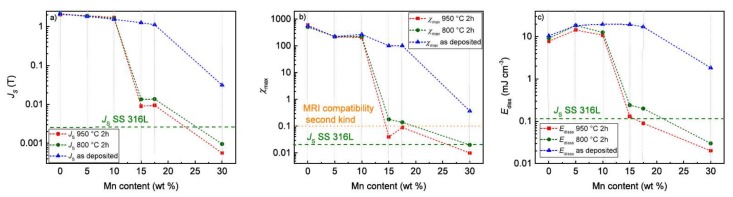
Graphic representation of the dependency between Mn content and (**a**) saturation polarization; (**b**) maximum susceptibility *χ*_max_; and (**c**) dissipated energy. The values of the SS 316 L reference are indicated by the green horizontal line. Additionally, in (**b**) the MRI compatibility according to Schenck [3] (given below) is also indicated, as a horizontal orange dot line.

In Table 6, the characteristic values for *J*_S_, *J*_R_, *μ*_0_*H*_C_, *χ*_max_, and *E*_diss_ are presented. The influence of the different Mn concentrations on *J*_S_, *χ*_max_, and *E*_dis*s*_ are graphically displayed in Figure 6. In general, the values of *J_S_*, *J_R_*, and *χ*_max_, of the as deposited samples decreased with increasing Mn content. In comparison, with the as-deposited samples, *J_S_*, *J_R_*, and *χ*_max_ decreased after annealing. However, at lower Mn concentrations (5/10 wt %), the values of the samples annealed at higher temperatures increased. Samples with higher Mn content (15–32 wt %) showed a decreasing tendency of *J*_S_, *J*_R_, and *χ*_max_ with increasing annealing temperature. The *E*_diss_ of the as-deposited samples first increased up to a Mn content of 10 wt %, and then stayed more or less constant. The FeMn32 showed a marked decrease of *E*_diss_*.* Annealing led to a decrease of *E*_diss_ compared to the as-deposited samples. At higher Mn concentrations (15/17 wt %), a marked drop of *E*_dis*s*_ was observed.

## 4. Discussion

### 4.1. Microstructure

It is shown that the presented approach is feasible for the fabrication of freestanding, in-situ structured Fe–FeMn composite films by using magnetron sputtering. From the SEM images, a clear separation of the layers was observed before annealing (Figure 3a). Based on the XRD measurements shown in Figure 2, as well as the previously-presented investigated microstructures of pure Fe [12] and FeMn32 [15], it is inferred that the pure Fe layers consisted of the ferritic α-Fe phase, while the FeMn32 layer was primarily composed of γ-FeMn, accompanied with small amounts of α-Fe. In contradiction to the results of the single layer FeMn32, even minor amounts of the ε-FeMn phase and α′-Mn phase are present, maybe related to the concentration shifts at the layer interfaces and the fact that a deposition by sputtering is not in thermodynamic equilibrium. Independent of the Mn content, after annealing the layer structure completely disappeared, and a recrystallized and homogeneous fine grained structure was observed. However, the Mn content showed a strong influence on the crystalline phase composition. According the XRD data, samples containing 5 wt % and 10 wt % Mn tended to increase the amount of α′-phase. However, the XRD data of the 800 °C annealed FeMn5/10 samples also showed evidence for the existence of the γ- and ε-phase. According to the XRD data of the annealed FeMn 15/17 samples, a stabilization of the ε- and γ-phase was observed. Nevertheless, due to the width of the overlapping reflexes, the α′(110) reflex cannot be assuredly excluded. Overall, the findings are in good agreement with the literature, and correlate with the non-equilibrium phase diagram of FeMn. The diagram predicts the formation of the α′-phase for Mn concentrations of 0–10 wt %. A phase mixture of α′-, ε-, and γ-phases is suggested for Mn concentrations of 10–14.5 wt %, while at concentrations of 14.5–27 wt % Mn, only ε- and γ-phases are expected [7,27]. The small grain sizes are attributed to the combination of different properties arising from the fabrication method. Sputtered films are known to exhibit a high defect density [35]. Also, the fabrication as Fe–FeMn multilayer may contribute to the grain size, since each layer interrupts a growing grain and provides additional nuclei. The combination of high defect densities and small initial grain sizes promotes recrystallization with a large number of additional nuclei, explaining the fine-grained microstructure after annealing. Furthermore, increasing the Mn content seems to have a grain-refining effect. In order to clearly distinguish between the influence of the grain size, the fabrication as multilayer, and the grain-refining effect of Mn, further studies would be necessary, in order to compare a multilayer system and single deposition of the same overall composition.

### 4.2. Mechanical Properties

The Mn content was found to strongly influence the mechanical properties. The FeMn5/10 samples both exhibited a high strength but low ductility. The high strength is, on one hand, attributed to the small grain size, in agreement with the Hall-Petch relation [36]; on the other hand, the strength is also attributed to the extensive solubility of the Mn atoms in the bcc Fe. With increasing Mn content, more Mn atoms are substituted for Fe atoms. The substituted Mn atoms induce internal stresses and hinder dislocation movement. As long as the α′-Fe phase is predominant, this results in an increasing strength and declining ductility at Mn concentrations 5/10 wt %. As discussed, higher amounts of Mn (15/17 wt %) lead to the stabilization of the ε- and γ-phase. These phases are known for showing the TRIP and TWIP effect. These effects occur due to the low stacking fault energy (SFE) of those alloys, which favor the formation of deformation twins or even a γ → ε phase transformation. Both effects are known to enhance mechanical strength and ductility, due to the extensive formation of deformation twins or strain-induced martensite. It is reported that both effects can coexist at lower Mn concentrations (23.8%). However, lower Mn concentrations favor the TRIP effect, whereas an intense strain-induced twinning, associated with the TWIP effect, is observed rather at higher Mn concentrations [37,38,39]. Therefore, it is assumed that at the Mn concentrations of 15/17 wt % used in this study, the TRIP effect is the predominant mechanism. The high strength of the FeMn15/17, compared to pure iron, is mainly attributed to the TRIP effect. The strength is even higher than that of FeMn32. One reason for this can be that the TRIP effect was found to contribute more to strength than the TWIP effect [37], which is predominantly seen in the FeMn samples. However, the smaller grains of the FeMn15/17 samples will increase the strength as well. The smaller grains of the FeMn17 sample also explains the higher YS in comparison to the FeMn15 samples. Since phase boundaries also hinder plastic deformation, the dual-phase microstructure contributes to the strength as well, and gives an additional reason for the higher strength compared to the strength of FeMn32, which exhibits an exclusively austenitic structure [15]. In general, the films exhibit a superior strength. With respect to the mechanical requirements (Table 5), the FeMn15 and FeMn17 samples exhibited the best properties, namely a very high strength accompanied by sufficient ductility. The strength of the samples exceeded the values given for the SS 316 L gold standard, and were even up to four times higher than the required values.

### 4.3. Corrosion

The initial trend of a decreasing corrosion rate (Figure 4) with increasing Mn content is found for electrochemical measurements, as well as for immersion tests. It was the origin of the idea [40] of using FeMn alloys in order to accelerate the corrosion of biodegradable Fe-based alloys. Since the *U*_0_ values shift to higher negative values with increasing Mn content, actually an increase of the corrosion rate was expected. However, the findings contradicted this expectation, and were in agreement with previous findings on sputtered FeMn32 [15]. The main decrease of CR already occurs at Mn concentrations of 5–10 wt %, which have a primarily ferritic structure. Thus, it is inferred that the major reason for the decrease is not the phase composition, but the Mn content. Studies on the influence of the electrolyte composition on the degradation behavior of FeMn alloys concluded that the amount of carbonate and hydrogen carbonate ions plays an essential role in the formation of insoluble MnCO_3_ layers, which can hinder further degradation [32,41]. The NaHCO_3_- and CO_2_-rich atmosphere was used to adjust the pH value, thus favoring the formation of those protection layers and explaining the low degradation rate for the Mn rich samples. Since the decrease of CR saturates at Mn concentrations >10 wt %, it is assumed that a critical Mn concentration (10–15 wt %) is necessary to reach the full protective effect. In general, degradation is a complex chemical process, and depends on a variety of parameters. Even though the in vitro test gave a hint, a direct comparison with the literature is difficult, due to different testing conditions. Furthermore, in vitro tests are not able to mimic the living system 100%, which makes in vitro tests indispensable. Even though a lower corrosion rate is a drawback with respect to the application, the higher strength allows the use of thinner structures, and in turn a higher relative surface, which reduces the retention time of an implant in the body. To make a definitive assessment of the materials’ suitability, in terms of the degradability, in vivo tests therefore should be performed in the future, even when considering geometrical design aspects.

### 4.4. Magnetic Properties

The magnetic properties (Figure 6 and Table 6) of the as-deposited samples mainly depend on the amount of FM α′-phase, where *J*_S_, *J*_R,_ and *χ*_max_ decrease with the Mn content and the amount of α′-phase. The increased *E*_diss_ of the FeMnX samples, compared to pure Fe, can be attributed to an FM–AFM pinning of the layers. This exchange anisotropy requires additional energy to rotate the magnetic moments, and is a well-known phenomenon [42]. The decrease of *J*_S_, *J*_R_, *χ*_max_, and *E*_diss_ with increasing Mn content is also, on one hand, related to the crystalline phase composition. On the other hand, different amounts of alloying elements will also affect the magnetic moment per atom, and in turn the magnetization. Paduani et al. showed that, when alloying up to 17 wt % Mn into bcc-stabilized Fe, a decrease of the magnetization with increasing Mn content is observed. The authors concluded that the effect is related to an antiparallel coupling of the magnetic moments, between the Fe and Mn atoms substituted as nearest neighbors. This decrease was described as nonlinear, with the most pronounced drop between 12% and 17% Mn [43]. Considering the results of microstructural analysis (Figure 2) at Mn concentrations <15 wt %, the decrease of *J*_S_ is related to the discussed mechanism. At higher Mn concentrations, the PM ε- and AFM γ-phases are primarily evident, explaining the low *J*_S_, *J*_R_, *χ*_max_, and *E*_diss_. Considering that, in terms of MRI compatibility, low values of *J*_S_, *χ*, and *E*_diss_ are required, the FeMn15/17 shows the best values. Furthermore, higher annealing temperatures facilitate low *J*_S_, *χ*, and *E*_diss_ values, probably due to the enhanced diffusion and, in turn, better homogenization. The values approach those of the SS 316 L standard. According to Schenk, the MRI compatibility of the first kind is defined as 10^−5^ < |*χ* − *χ*_water_| < 10^−2^, where *χ*_water_ = −9.05 × 10^−6^ [3]. According to this definition, the MRI compatibility is given for the FeMn15 and FeMn17 samples annealed at 950 °C.

## 5. Conclusions

It is shown that freestanding, in situ, structured Fe–FeMn32 multilayer films can be deposited via magnetron sputtering. By post-deposition annealing, the films can be homogenized, and a recrystallized, very fine-grained microstructure is obtained. The phase composition is influenced by the Mn content. At up to 10 wt %, the Mn α′-phase is present, whereas at Mn concentrations of 15 and 17 wt %, the γ- and ε-phase are observed. The Mn content significantly influences the material properties, summarized in the following:

- FeMn5: Showed a decrease of the ferromagnetic behavior and a decrease of the CR compared to pure Fe—a high strength, but a rather low ductility
- FeMn10: Further decrease of the ferromagnetic behavior and a further decrease of CR compared to FeMn5—an increase of strength, but lower ductility.
- FeMn15: Marked drop of ferromagnetic characteristics and a slightly decreased CR compared to FeMn10—lower but still high strength, with enhanced ductility.
- FeMn17: Similar magnetic and corrosion behavior to FeMn15, with a higher YS but similar UTS and ductility, compared to FeMn15.

With respect to the desired application, the FeMn15 and FeMn17 showed great potential and the best compromise between high strength, sufficient ductility, non-ferromagnetic behavior, and low Mn content. The samples distinctly exceeded the requirements in terms of the mechanical properties. Even the magnetic properties were very promising, and show that Mn concentrations of 15/17 wt % are sufficient in order to reach MRI compatibility of the first kind. The low degradation rate could be compensated for by increasing the relative surface of an implant. Thus, fabrication by magnetron sputtering is clearly beneficial, due to the fine-grained microstructure and in turn, the high strength that can be achieved. Furthermore, the small feature sizes that can be achieved by the in-situ structuring give a large freedom of design and make formative processing steps obsolete, which could affect the microstructure and material properties.

## Figures and Tables

**Table 1:** Sample parameters.

Sample	Target (s)	FeMn32 Thickness (nm)	Nominal Mn Content (wt %)
Fe [12]	pure Fe	-	0.0
FeMn5	Fe-FeMn35 multilayer	50	4.8
FeMn10	Fe-FeMn35 multilayer	125	10.0
FeMn15	Fe-FeMn35 multilayer	250	15.5
FeMn17	Fe-FeMn35 multilayer	325	17.2
FeMn32 [15]	Pre-alloyed FeMn35	-	32

Fe layer thickness constant (250 nm) for all ML samples.

**Table 2:** List of relevant 2θ angles, corresponding phases and lattice planes.

Symbol	2θ (°)	hkl
α′ 1	44.660	110
α′ 2	65.000	200
α′ 3	82.300	211
γ 1	43.120	111
γ 2	50.220	200
γ 3	73.760	220
γ 4	89.460	311
ε 1	41.160	100
ε 2	44.380	002
ε 3	47.040	101
ε 4	62.120	102
ε 5	75.020	110
ε 6	83.640	103
ε 7	89.360	200
ε 8	91.540	112

**Table 3:** Grain sizes.

Sample	$\bar{d}$ (μm)	*d_min_* (μm)	*d_max_* (μm)
Fe 800 °C, 2 h [12]	3.01	-	-
FeMn5 800 °C, 2 h	0.621	0.062	2.139
FeMn10 800 °C, 2 h	0.521	0.093	1.731
FeMn15 800 °C, 2 h	0.481	0.076	1.497
FeMn17 800 °C, 2 h	0.406	0.078	1.133
FeMn32 800 °C, 2 h [15]	0.965	3.193	0.068
FeMn5 950 °C, 2 h	0.965	0.223	3.478
FeMn10 950 °C, 2 h	0.860	0.179	2.208
FeMn15 950 °C, 2 h	0.843	0.216	2.635
FeMn17 950 °C, 2 h	0.578	0.990	1.618
FeMn32 950 °C, 2 h [15]	1.305	5.078	0.143

**Table 4:** Mean values of *U*_0_, *j*_0_, corrosion rate (CR), and weight loss (WL), determined from the electrochemical and immersion corrosion measurements.

Sample	*U*_0_ (V)	*j*_0_ (A/m²)	CR (mm/year)	WL (mg/cm²)
Fe 800 °C, 2 h [12,14]	−0.661 ± 0.019	0.092 ± 0.008	0.101 ± 0.009	3.80 ± 0.84
Fe 950 °C, 2 h	−0.657 ± 0.025	0.109 ± 0.025	0.120 ± 0.012	3.01 ± 0.54
FeMn5 800 °C, 2 h	−0.705 ± 0.007	0.651 ± 0.010	0.072 ± 0.006	3.17 ± 1.01
FeMn5 950 °C, 2 h	−0.704 ± 0.015	0.073 ± 0.006	0.081 ± 0.007	2.25 ± 0.48
FeMn10 800 °C, 2 h	−0.724 ± 0.006	0.053 ± 0.005	0.059 ± 0.006	2.34 ± 0.99
FeMn10 950 °C, 2 h	−0.719 ± 0.004	0.064 ± 0.007	0.071 ± 0.006	1.98 ± 0.64
FeMn15 800 °C, 2 h	−0,735 ± 0,008	0.036 ± 0.006	0.040 ± 0.018	1.65 ± 0.63
FeMn15 950 °C, 2 h	−0.728 ± 0.008	0.038 ± 0.019	0.042 ± 0.008	1.49 ± 0.45
FeMn17 800 °C, 2 h	−0.737 ± 0.018	0.034 ± 0.008	0.030 ± 0.008	1.41 ± 0.32
FeMn17 950 °C, 2 h	−0.737 ± 0,019	0.030 ± 0.002	0.033 ± 0.002	1.42 ± 0.35
FeMn32 800 °C, 2 h [15]	−0.749 ± 0.006	0.041 ± 0.011	0.045 ± 0.012	1.81 ± 0.65
FeMn32 950 °C, 2 h [15]	−0.741 ± 0.010	0.048 ± 0.010	0.054 ± 0.011	1.68 ± 0.75

**Table 5:** Mean values and standard deviations of the mechanical properties for the different FeMnX samples after annealing. Additionally, the values for sputtered pure Fe [12] and FeMn32 are given [15], as well as the SS 316 L gold standard [10] and required reference values for the desired application.

Sample	YS (MPa)	UTS (MPa)	A (%)
Fe 800 °C, 2 h [12]	266.9 ± 7.5	343.8 ± 4.8	20.0 ± 2.7
Fe 950 °C, 2 h	191.3 ± 34.7	246.6 ± 7.3	4.0 ± 0.8
FeMn5 800 °C, 2 h	686.2 ± 32.0	918.2 ± 44.6	4.2 ± 0.6
FeMn5 950 °C, 2 h	686.3 ± 24.1	809.1 ± 9.7	5.6 ± 1.0
FeMn10 800 °C, 2 h	905.8 ± 21.3	1242.9 ± 36.2	2.8 ± 0.5
FeMn10 950 °C, 2 h	805.3 ± 35.3	1100.9 ±11.5	2.2 ± 0.5
FeMn15 800 °C, 2 h	766.6 ± 27.1	1080.7± 20.9	17.0 ± 1.1
FeMn15 950 °C, 2 h	697.4 ± 59.7	963.9 ± 41.8	20.5 ± 2.4
FeMn17 800 °C, 2 h	824.8 ± 27.0	1061.4 ± 29.4	21.5 ± 1.7
FeMn17 950 °C, 2 h	857.0 ± 18.8	984.7 ± 22.9	18.5 ± 4.6
FeMn32 800 °C, 2 h [15]	571.0 ± 11.5	712.5 ± 3.7	23.1 ± 1.6
FeMn32 950 °C, 2 h [15]	541.0 ± 10.9	678.9 ± 15.9	24.4 ± 1.7
SS 316 L [10,33]	190	490	40
Required properties [34]	>200	>300	>15–18

**Table 6:** Magnetic properties.

Sample	*J*_S_ (T)	*J*_R_ (T)	*μ*_0_*H*_C_ (mT)	*χ*_max_	*E*_diss_ (mJ/cm^3^)
Fe as deposited [15]	2.206	0.650	1.874	560.59	10.50
Fe 800 °C, 2 h [15]	2.141	0.545	1.695	510.26	9.10
Fe 950 °C, 2 h [15]	2.052	0.715	1.147	595.34	7.77
FeMn5 as deposited	1.822	0.462	2.115	225.18	18.32
FeMn5 800 °C, 2 h	1.888	0.340	1.463	223.26	18.35
FeMn5 950 °C, 2 h	1.919	0.391	1.804	218.45	14.30
FeMn10 as deposited	1.542	0.682	2.62	270.22	19.59
FeMn10 800 °C, 2 h	1.619	0.486	2.530	221.57	12.59
FeMn10 950 °C, 2 h	1.726	0.395	1.876	207.46	10.83
FeMn15 as deposited	1.257	0.360	3.699	101.92	19.38
FeMn15 800 °C, 2 h	0.014	1.12 × 10^−3^	8.440	0.18	0.24
FeMn15 950 °C, 2 h	9.31 × 10^−3^	0.38 × 10^−3^	12.549	0.04	0.13
FeMn17 as deposited	1.118	0.289	2.959	102.41	17.04
FeMn17 800 °C, 2 h	0.014	0.84 × 10^−3^	8.026	0.14	0.20
FeMn17 950 °C, 2 h	9.40 × 10^−3^	0.13 × 10^−3^	7.129	0.09	0.09
FeMn32 as deposited [15]	0.032	6.02 × 10^−3^	26.32	0.37	1.83
FeMn32 800 °C, 2 h [15]	1.09 × 10^−3^	0.13 × 10^−3^	14.752	0.02	0.03
FeMn32 950 °C, 2 h [15]	0.64 × 10^−3^	0.04 × 10^−3^	8.586	0.01	0.02
SS 316L [15]	2.65 × 10^−3^	0.51 × 10^−3^	30.212	0.02	0.11
